# Comparing Usual Care With Coordinated Clinician and Patient Use of Mobile Technology in Primary Care for Patients With Major Depressive Disorder: Practice-Based Pilot Study

**DOI:** 10.2196/71662

**Published:** 2026-05-06

**Authors:** Angela M Lanigan, Elisabeth F Callen, Melissa K Filippi, Elise A Robertson, Tarin L Clay, Andrea Nederveld, Ben Fehnert, Lambros Chrones, Michael L Martin, Maggie McCue, Christina M Hester

**Affiliations:** 1 National Research Network, American Academy of Family Physicians, Leawood, KS, United States; 2 DARTNet Institute, 12635 East Montview Blvd, Suite 129, Aurora, CO, 80045, United States, 1-800-434-0278 ext 78; 3 Robert Graham Center, American Academy of Family Physicians, Washington, DC, United States; 4 Department of Family Medicine, University of Colorado Anschutz Medical Campus, Aurora, CO, United States; 5 Ctrl Group/Fora Health, London, United Kingdom; 6 Takeda Pharmaceuticals USA, Inc, Lexington, MA, United States

**Keywords:** goal setting, major depressive disorder, MDD, mobile app technology, practice-based research, primary care, shared decision-making, digital health

## Abstract

**Background:**

Major depressive disorder (MDD) affects millions of Americans each year and is often diagnosed and treated in primary care. Evidence shows that self-management techniques, shared decision-making (SDM), and goal setting are effective strategies for managing MDD, but the required collaboration between patients and primary care clinicians can be difficult. Primary Care Path is a program for supporting depression management in primary care that includes a patient-facing mobile app and an accompanying care team–facing web interface. Leveraging programs that provide clinician-facing software with companion patient-facing mobile technology may help patients and physicians align depression treatment and management goals, support effective SDM, alleviate barriers, and improve both clinical care and patient outcomes.

**Objective:**

To pilot-test the use of Primary Care Path for MDD management in primary care and evaluate the impact of its use on depression treatment, symptoms, goal setting and attainment, and SDM.

**Methods:**

Four primary care clinical practices in the United States were assigned to program use (2 practices; intervention) versus usual care (2 practices; control). Intervention practices used the Primary Care Path program in their clinics and engaged patient participants in app use for 18 weeks. Clinical care teams engaged with the patient-informed program portal primarily during patient encounters (in-person, virtual or calls). Patient participants were smartphone users aged 18 years and older who were being treated for MDD. Patient participants received online surveys (medication changes, Patient Health Questionnaire-9 [PHQ-9], goal setting and attainment questions, and Shared Decision-Making Questionnaire-9 [SDM-Q-9]) at baseline, 6, 12, and 18 weeks.

**Results:**

A total of 76 patient participants (34 intervention; 42 control) were enrolled; the majority were female (27/34, 79%; 32/42, 76%), White (31/34, 91%; 40/42, 95%), non-Hispanic/Latino/a (29/34, 85%; 40/40, 100%), and employed (26/34, 77%; 34/42, 81%). Control patient participants’ conversations with their medical providers increased over the study period, while intervention patient conversations with their medical providers decreased over time. At week 18, intervention participants felt more successful than control in achieving their personalized treatment goals. More intervention patient participants initiated antidepressant medication by weeks 12 (*P*=.03) and 18 (*P*=.04) and switched medications by weeks 6 (*P*=.009) and 12 (*P*=.04) versus control. All patient participants demonstrated significant improvement in PHQ-9 scores throughout the study period (*P*<.001), with no difference in change by group. Clinicians and patients indicated using the program to support SDM, but no significant differences were observed in SDM-Q-9 between intervention and control.

**Conclusions:**

Preliminarily, the use of this digital health program related to earlier medication optimization, earlier conversations between patients and medical providers, and patient attainment of goals that matter most to them, indicating that coordinated use of the program by both patients and clinical team members may enhance MDD management in primary care clinical settings.

## Introduction

Major depressive disorder (MDD) affects approximately 8% of Americans each year [1], with 21 million Americans reporting having a major depressive episode in the past year in 2020 [2]. Effective treatments for MDD include medication and counseling or therapy. Self-management and goal setting are also associated with improved outcomes for people with MDD [3].

Primary care clinicians play an important role in engaging patients to set individual and personal goals for treatment and in supporting goal attainment and yielding improved outcomes for patients with MDD [4,5]. However, research shows that primary care clinical teams face challenges around goal setting as a component of depression management compared with similarly prevalent physical ailments [6].

Software apps, particularly those accessible on mobile devices, have increased in popularity and availability over the past decade, with more than 10,000 marketed for mental health support [7], often used to assist in goal setting and self-management for many conditions and circumstances. Digital resources are emerging as an important tool for both patients and clinicians to optimize their communication and lead to improved outcomes, but their efficacy needs to be further explored [8,9]. Furthermore, data sharing by patients and use by clinicians is an important strategy for care optimization and may prompt patients to engage clinicians when concerns arise [10]; however, the optimal strategies for incorporating the varying types of app-collected patient data into clinical care must be further explored. Preliminary evidence suggests that apps may be effective tools for supporting communication and serving as a resource to the shared decision-making (SDM) process in the primary care setting for MDD [11-15].

Here, we report on small-scale pilot-testing of a program called Primary Care Path (Fora Health), designed to improve patient self-management of MDD through goal setting, daily tracking of medication and side effects, and weekly assessments of depression severity, well-being, and goal attainment. Program collaborators consisting of the research team, Takeda Pharmaceuticals, and Fora Health all contributed to the development of the program for this study. The program consisted of an app for patients that interfaced with a clinician-facing web portal where clinicians could access, review, and monitor participants’ app-entered data during or between encounters (in-person or virtual visits or calls), as well as view graphs and charts of longitudinal data for each enrolled participant. During the pilot study, clinicians were not expected to review app data daily but were asked to use or refer to them, as appropriate, to support SDM during encounters.

## Methods

### Recruitment

#### Practices

Primary care practices with behavioral health integrated into their clinic teams and structures, which provide treatment for patients with depression and regularly administer the Patient Health Questionnaire-9 (PHQ-9) [16,17], and were members of the American Academy of Family Physicians National Research Network in 2020 recruited via email. Four practices were assigned (nonrandomized, practice-level pilot) into the study (2 intervention and 2 control). However, due to circumstances related to the COVID pandemic, 1 intervention site withdrew before onboarding but after the other 3 sites had been onboarded. This practice was replaced by recruiting another practice directly into the intervention arm. Participating practices were located in Michigan (n=1), Florida (n=1), and New York (n=2), and were not blinded as to which group they were assigned. Practice teams were trained to support patient referral for consent and enrollment by the study team, and practices in both arms received facilitation support for study-related activities from the study team throughout the implementation period.

#### Participants

Patient participants met the following criteria: ≥18 years of age, PHQ-9 score >5, being treated or initiating antidepressant monotherapy or behavioral health, owned a smartphone, and had no co-occurring major psychiatric disorder, reading or clinically significant learning disability, or recent hospitalization or suicide attempt. Participants were recruited from among patients with recent (past 6 weeks) or upcoming appointments for depression treatment. Patient participants were enrolled in the study on a rolling basis (participating for 18 weeks per participant) from November 2020 through June 2021.

### Ethical Considerations

This study was approved by the American Academy of Family Physicians Institutional Review Board (protocol 20‐389) under categories 5 and 7. Participants were enrolled and consented electronically via a waiver of documentation of consent process. Participant confidentiality was protected by storage of data within a secure information technology environment and maintained as participant identifiers were removed for analysis. Patients received e-gift cards in the amounts of US $30 for completing the enrollment survey, US $25 for each of the 4 follow-up surveys, and were given a US $40 bonus if all 4 follow-up surveys were completed on time. E-gift cards were sent via an automated email after the completion of the survey from RewardsGenius/Tango Card.

### Intervention

Intervention practices used the Primary Care Path program, which consisted of a patient-facing mobile app and accompanying care team website (subsequently referred to as “the program”) with their participants to facilitate SDM, symptom tracking, and medication adherence. Practices in the control group did not use the program but recruited patients to respond to study surveys over an 18-week period. The app allowed clinician entry of goals created by patients in conversation with their clinician. Patient participants were able to track medication adherence (daily) and mental and physical symptoms on a daily or weekly basis. Goal setting was undertaken with clinical care team members, and goal attainment was tracked on a weekly basis within the app. Clinical care team members at intervention practices were able to see up-to-date information from patient app use when they accessed the program.

### Data Collection

#### Research Survey

Participants in both groups (intervention and control) completed online research surveys administered through Qualtrics (Qualtrics, LLC) assessing SDM (Shared Decision-Making Questionnaire -9 [SDM-Q-9]) [18], depression symptoms (PHQ-9), treatment decisions (ie, starting or stopping a medication, increasing or decreasing medication dose, etc), and goal setting or progress at baseline, 6, 12, and 18 weeks. The SDM-Q-9 was used to evaluate patient perception of the shared decision-making process and asked questions such as “My doctor made clear a decision needs to be made,” and “My doctor and I thoroughly weighed the different treatment options” [18]. Patients were sent automated invites when it was time for them to complete surveys and reminders every 2-3 days until completion or until 2 weeks had passed.

#### App Use and Engagement Data

Data on app use were collected by the app developer and shared with the study team for analysis. App engagement was dichotomized after review of usage data at the end of the study into High Engagers and Low Engagers. The cut point was established post hoc based on identification of the natural cut point in the usage data (less than 7 interactions with the app; significant gap in interactions with the app).

### Statistical Analysis

Descriptive statistics (counts, percentages, means, and standard deviations, as appropriate) were calculated for all questions and scores. Exploratory analyses consisted of chi-square tests (dichotomous variables), ANOVAs (categorical variables more than 2 categories), and ANCOVAs (controlling for demographic variables) were used for inferential statistics to determine single time point results using the full data at each time point (group results; separate analyses were performed for High Engagers as well as for all Intervention against Control). For additional exploratory analyses, mixed ANOVAs were used for analyses over time and used data at the individual level; an individual had to have data at each time point to be included in the analysis. Mixed ANOVAs, which require all data to be present, were performed for the following combinations: (1) baseline to 6 weeks, (2) baseline to 12 weeks, (3) baseline to 18 weeks, (4) baseline to 6-12 weeks, and (5) baseline to 6 weeks to 12 weeks to 18 weeks. Missing data were removed casewise for each analysis. Tukey HSD and Games-Howell were used for post hoc analyses. For all analyses, all data collected were included. However, for the tests that were tracking a person over time (ie, mixed ANOVA), the respondent had to complete each assessment included in the analysis. The mixed ANOVA was a per-protocol analysis. While these were exploratory analyses due to the pilot or feasibility nature of the study, we did apply the Bonferroni correction for each comparison and noted where it did and did not affect results. An α value of .05 for 2-sided tests was used for statistical significance. SPSS (version 27; IBM Corp) was used for all analyses.

## Results

### App Use and Engagement

Intervention patients used the app with a wide range of engagement. Interaction touchpoints ranged from 0 to 126 throughout the course of a participant’s involvement in the study. App use data yielded 2 levels of app engagement among intervention participants. A natural cutoff in the data was found with <7 app touchpoints corresponding to low engagement and ≥7 touchpoints indicating high engagement (“High Engagers”). This natural cutoff also corresponded to intervention patients interacting with the app for less than 2 weeks (data not shown). The High Engagers group was established as a post hoc stratification once it became clear that there were 2 distinct levels of usage among the intervention patients.

### Demographics

The majority of the participants recruited were female (Control: 32/42, 76.2%; All Intervention: 27/34, 79.4%; and High Engagers: 22/27, 81.5%) and White (Control: 40/42, 95.2%; All Intervention: 31/34, 91.2%; and High Engagers: 22/27, 92.6%; Table 1). Participants were equally distributed in relation to gender and race across groups (gender: Control/All Intervention: *P*=.95, Control/High Engagers: *P*≥.99; race: Control/All Intervention: *P*=.43, Control/High Engagers: *P*=.70). However, there were Hispanic/Latinx participants in the All Intervention/High Engagers group but none in the Control group (Control: 0.0%, 0/42; All Intervention: 14.7%, 5/34; High Engagers: 18.5%, 5/27; Control/All Intervention: *P*=.01, Control/High Engagers: *P*=.003). Other demographic variables (eg, highest degree, marital status, type of health insurance, and income) did not differ between groups and are reported in Table 1.

**Table 1.** Study participant characteristics by group.

Characteristics	Control (n=42), n (%)	All intervention (n=34), n (%)	High engagers (n=27),n (%)	*P* value
Sex				
Male	8 (19.0)	7 (20.6)	5 (18.5)	C^a^/A^b^: .95 C/H^c^: ≥.99
Female	32 (76.2)	27 (79.4)	22 (81.5)	
Missing	2 (2.8)	0 (0.0)	0 (0.0)	
Race				
White	40 (95.2)	31 (91.2)	25 (92.6)	C/A: .43 C/H: .70
Black or African American	1 (2.4)	2 (5.9)	1 (3.7)	
Missing	1 (2.4)	1 (2.9)	1 (3.7)	
Ethnicity				
Hispanic or Latinx	0 (0.0)	5 (14.7)	5 (18.5)	C/A: .01^d^ C/H: .003^d^
Non-Hispanic or Latinx	40 (95.2)	29 (85.3)	22 (81.5)	
Missing	2 (4.8)	0 (0.0)	0 (0.0)	
Education				
High school or less	15 (35.7)	8 (23.5)	5 (18.5)	C/A: .24 C/H: .10
Through Associate’s degree	19 (45.2)	15 (44.1)	13 (48.1)	
Bachelor’s degree and above	7 (16.7)	11 (32.4)	9 (33.3)	
Missing	1 (2.4)	0 (0.0)	0 (0.0)	
Marital Status				
Single, divorced, or widowed	29 (69.0)	17 (50.0)	14 (51.9)	C/A: .07 C/H: .13
Married or separated	12 (28.6)	17 (50.0)	13 (48.1)	
Missing	1 (2.4)	0 (0.0)	0 (0.0)	
Employment				
Full or part time	34 (81.0)	26 (76.5)	21 (77.8)	C/A: .33 C/H: .63
Unemployed	5 (11.9)	3 (8.8)	3 (11.1)	
Other	2 (4.8)	5 (14.7)	3 (11.1)	
Missing	1 (2.4)	0 (0.0)	0 (0.0)	
Insurance				
Medicare/Medicaid	20 (47.6)	11 (32.4)	14 (51.9)	C/A: .15 C/H: .18
Private/Other	21 (50.0)	23 (67.6)	13 (48.1)	
Missing	1 (2.4)	0 (0.0)	0 (0.0)	
Selected treatments				
Antidepressant medication	33 (78.6)	27 (79.4)	23 (85.2)	C/A: .91 C/H: .72
Behavioral health/talk therapy	20 (47.6)	9 (26.5)	8 (29.6)	C/A: .048 C/H: .18

a C: control.

b A: all intervention.

c H: high engagers.

d *P*<.05.

### Results That Remain Significant After Correcting for Multiple Comparisons

#### Changes in Depression Symptoms

At the survey time points after baseline, all participant groups had significant decreases in their PHQ-9 score, indicating that all participants saw an improvement in their depression symptoms. However, there was no significant difference between any of the participant groups at any point in time or over time (Table 2).

**Table 2.** Comparisons between groups over time for both All Intervention versus Control and High Engagers versus Control.

Question	Control, mean (SD)	All Intervention, mean (SD)	High Engagers, mean (SD)	Control/All Intervention Test, *F* test (*df*)	Control/High Engagers Test
SDM-Q-9^a^ score	0: 84.06 (16.25)	0: 79.09 (18.77)	0: 81.06 (17.21)	Time: *F* (1, 60)=3.060; *P*=.09	Time: *F* (1, 56)=4.527; *P*=.04
	6: 77.90 (20.20)	6: 75.06 (23.32)	6: 74.30 (26.72)	Time × Group: *F* (1, 60)=0.133; *P*=.72	Time × Group: *F* (1, 56)=0.010; *P*=.92
	0: 85.14 (16.24)	0: 77.69 (18.65)	0: 78.35 (21.45)	Time: *F* (1, 58)=3.748; *P*=.06	Time: *F* (1, 54)=8.220; *P*=.006
	12: 78.35 (21.45)	12: 75.73 (21.92)	12: 73.12 (22.29)	Time × Group: *F* (1, 58)=1.146; *P*=.29	Time × Group: *F* (1, 54)=0.021; *P*=.89
	0: 86.46 (11.88)	0: 79.32 (19.10)	0: 80.53 (17.45)	Time: *F* (1, 57)=6.075; *P*=.02	Time: *F* (1, 52)=6.559; *P*=.01
	18: 76.70 (20.18)	18: 75.21 (20.22)	18: 75.34 (20.96)	Time × Group: *F* (1, 57)=1.013; *P*=.32	Time × Group: *F* (1, 52)=0.615; *P*=.44
PHQ-9^b^ score	0: 13.86 (5.77)	0: 13.25 (5.41)	0: 12.83 (5.29)	Time: *F* (1, 61)=31.600; *P*<.001	Time: *F* (1, 57)=26.231; *P*<.001
	6: 10.46 (4.94)	6: 9.18 (5.77)	6: 9.54 (6.12)	Time × Group: *F* (1, 61)=0.255; *P*=.62	Time × Group: *F* (1, 57)=0.007; *P*=.93
	0: 13.74 (5.71)	0: 12.85 (5.24)	0: 12.23 (5.71)	Time: *F* (1, 59)=35.929; *P*<.001	Time: *F* (1, 55)=29.005; *P*<.001
	12: 9.09 (5.73)	12: 8.19 (5.91)	12: 8.32 (6.36)	Time × Group: *F* (1, 59)=0.000; *P*≥.99	Time × Group: *F* (1, 55)=0.221; *P*=.64
	0: 13.70 (5.75)	0: 13.36 (5.18)	0: 12.95 (5.25)	Time: *F* (1, 59)=21.948; *P*<.001	Time: *F* (1, 53)=20.924; *P*<.001
	18: 10.24 (5.75)	18: 9.39 (6.95)	18: 9.23 (7.25)	Time × Group: *F* (1, 59)=0.104; *P*=.75	Time × Group: *F* (1, 53)=0.030; *P*=.86
	0: 13.88 (5.84)	0: 12.88 (5.34)	0: 12.23 (5.04)	Time: *F* (1, 56)=22.683; *P*<.001	Time: *F* (1, 53)=18.110; *P*<.001
	6: 10.33 (4.99)	6: 9.28 (5.85)	6: 9.59 (6.13)	Time × Group: *F* (1, 56)=0.026; *P*=.98	Time × Group: *F* (1, 53)=0.201; *P*=.82
	12: 9.42 (5.70)	12: 8.12 (6.02)	12: 8.32 (6.36)	N/A^c^	N/A
	0: 13.86 (5.98)	0: 13.17 (5.26)	0: 12.52 (4.97)	Time: *F* (3, 49)=17.708; *P*<.001	Time: *F* (1, 48)=14.081; *P*<.001
	6: 10.21 (5.02)	6: 9.21 (5.96)	6: 9.92 (5.23)	Time × Group: *F* (3, 49)=0.328; *P*=.81	Time × Group: *F* (1, 48)=0.260; *P*=.85
	12: 8.93 (5.23)	12: 8.21 (6.13)	12: 8.43 (6.49)	N/A	N/A
	18: 10.62 (5.63)	18: 8.67 (7.06)	18: 9.00 (7.35)	N/A	N/A
Decision: Switched antidepressant medication^d^	0: 0.09 (0.28)	0: 0.07 (0.27)	0: 0.09 (0.29)	Time: *F* (1, 60)=1.431; *P*=.24	Time: *F* (1, 56)=2.042; *P*=.16
	6: 0.03 (0.17)	6: 0.22 (0.42)	6: 0.26 (0.45)	Time × Group: *F* (1, 60)=7.281; *P*=.009	Time × Group: *F* (1, 56)=7.995; *P*=.006
	0: 0.09 (0.29)	0: 0.08 (0.28)	0: 0.10 (0.30)	Time: *F* (2, 54)=1.562; *P*=.21	Time: *F* (1, 52)=1.773; *P*=.18
	6: 0.03 (0.17)	6: 0.25 (0.44)	6: 0.29 (0.46)	Time × Group: *F* (2, 54)=3.232; *P*=.04	Time × Group: *F* (1, 52)=3.454; *P*=.04
	12: 0.00 (0.00)	12: 0.13 (0.34)	12: 0.14 (0.36)	N/A	N/A
Decision: Started an antidepressant medication^d^	0: 0.31 (0.47)	0: 0.39 (0.50)	0: 0.40 (0.50)	Time: *F* (1, 50)=3.905; *P*=.01	Time: *F* (1, 47)=3.817; *P*=.01
	6: 0.34 (0.48)	6: 0.39 (0.50)	6: 0.45 (0.51)	Time × Group: *F* (1, 50)=0.871; *P*=.46	Time × Group: *F* (1, 47)=0.624; *P*=.60
	12: 0.10 (0.31)	12: 0.35 (0.49)	12: 0.35 (0.49)	N/A	N/A
	18: 0.07 (0.26)	18: 0.26 (0.45)	18: 0.20 (0.47)	N/A	N/A
Decision: Discontinued an antidepressant medication^d^	0: 0.00 (0.00)	0: 0.04 (0.21)	0: 0.00 (0.00)	Time: *F* (1, 50)=1.992; *P*=.12	Time: *F* (1, 47)=3.285; *P*=.02
	6: 0.03 (0.19)	6: 0.00 (0.00)	6: 0.00 (0.00)	Time × Group: *F* (1, 50)=1.992; *P*=.12	Time × Group: *F* (1, 47)=2.071; *P*=.11
	12: 0.10 (0.31)	12: 0.00 (0.00)	12: 0.00 (0.00)	N/A	N/A
	18: 0.07 (0.26)	18: 0.13 (0.34)	18: 0.15 (0.37)	N/A	N/A
Past 18 weeks, set any treatment goals for your depression^e^	0: 1.43 (0.50)	0: 1.44 (0.51)	0: 1.50 (0.51)	Time: *F* (1, 58)=5.864; *P*=.02	Time: *F* (1, 55)=8.968; *P*=.004
	6: 1.34 (0.48)	6: 1.20 (0.41)	6: 1.18 (0.395)	Time × Group: *F* (1, 58)=1.316; *P*=.26	Time × Group: *F* (1, 55)=2.971; *P*=.09
	0: 1.38 (0.49)	0: 1.46 (0.51)	0: 1.55 (0.51)	Time: *F* (1, 56)=5.224; *P*=.03	Time: *F* (1, 52)=5.987; *P*=.02
	12: 1.32 (0.48)	12: 1.21 (0.42)	12: 1.25 (0.44)	Time × Group: *F* (1, 56)=2.002; *P*=.16	Time × Group: *F* (1, 52)=2.705; *P*=.11
	0: 1.34 (0.48)	0: 1.42 (0.50)	0: 1.50 (0.51)	Time: *F* (1, 56*)*=3.402; *P*=.07	Time: *F* (1, 50)=6.653; *P*=.01
	18: 1.34 (0.48)	18: 1.15 (0.37)	18: 1.10 (0.31)	Time × Group: *F* (1, 56)=3.402; *P*=.07	Time × Group: *F* (1, 50)=6.653; P=.01
	0: 1.41 (0.50)	0: 1.48 (0.51)	0: 1.55 (0.51)	Time: *F* (1, 53)=4.057; *P*=.02	Time: *F* (1, 50)=5.531; *P*=.005
	6: 1.31 (0.47)	6: 1.22 (0.42)	6: 1.20 (0.41)	Time × Group: *F* (1, 53)=1.197; *P*=.31	Time × Group: *F* (1, 50)=2.022; *P*=.14
	12: 1.34 (0.48)	12: 1.22 (0.42)	12: 1.25 (0.44)	N/A	N/A
	0: 1.36 (0.49)	0: 1.45 (0.51)	0: 1.53 (0.51)	Time: *F* (1, 48)=1.744; *P*=.16	Time: *F* (1, 45)=2.813; *P*=.04
	6: 1.29 (0.46)	6: 1.23 (0.43)	6: 1.21 (0.42)	Time × Group: *F* (1, 48)=1.838; *P*=.13	Time × Group: *F* (1, 45)=2.918; *P*=.04
	12: 1.32 (0.48)	12: 1.23 (0.43)	12: 1.26 (0.45)	N/A	N/A
	18: 1.39 (0.50)	18: 1.14 (0.35)	18: 1.11 (0.32)	N/A	N/A
Past 18 weeks, how successful have you felt in achieving your treatment goal(s) for your depression?^e^	0: 2.56 (0.88)	0: 2.19 (1.03)	0: 2.12 (1.05)	Time: *F* (1, 51)=13.729; *P*=.001	Time: *F* (1, 45)=14.072; *P*<.001
	18: 2.84 (0.72)	18: 3.05 (0.87)	18: 3.06 (0.90)	Time × Group: *F* (1, 51*)*=3.513; *P*=.07	Time × Group: *F* (1, 45)=4.101; *P*=.049
	0: 2.50 (0.88)	0: 2.24 (1.03)	0: 2.13 (1.06)	Time: *F* (1, 43)=7.436; *P*<.001	Time: *F* (1, 41)=7.236; *P*<.001
	6: 2.86 (0.65)	6: 2.65 (0.93)	6: 2.60 (0.99)	Time × Group: *F* (1, 43)=2.703; *P*=.048	Time × Group: *F* (1, 41)=2.698; *P*=.049
	12: 2.89 (0.63)	12: 3.06 (0.90)	12: 3.00 (0.93)	N/A	N/A
	18: 2.75 (0.70)	18: 3.24 (0.75)	18: 3.20 (0.78)	N/A	N/A
Past 18 weeks, discussed depression treatment goals with your medical provider^e^	0: 1.15 (0.36)	0: 1.22 (0.42)	0: 1.24 (0.44)	Time: *F* (1, 58)=0.057; *P*=.81	Time: *F* (1, 52)=0.298; *P*=.59
	18: 1.27 (0.45)	18: 1.07 (0.27)	18: 1.05 (0.22)	Time × Group: *F* (1, 58)=5.721; *P*=.02	Time × Group: *F* (1, 52)=6.029; *P*=.02

a SDM-Q-9: Shared Decision-Making Questionnaire-9.

b PHQ-9: Patient Health Questionnaire-9.

c N/A: not applicable.

d 0 = No, 1 = Yes.

e 1 = Unsuccessful, 2 = Somewhat unsuccessful, 3 = Somewhat successful, and 4 = Very successful.

### Goal Setting and Progress and Communication

No matter the time elapsed, all participants saw significant increases in feeling successful in achieving their treatment goals for depression. Between baseline and 18 weeks, the High Engagers group had a significantly larger increase in feeling successful than the Control group (Time: *F*_1, 45_=14.072, *P*<.001; Time × Group: *F*_1, 45_=4.101, *P*=.049). When including all time points over the study period, the High Engagers and All Intervention groups had significantly larger increases in feeling successful than the Control group (Control/All Intervention: Time: *F*_3, 123_=7.436, *P*<.001, Time × Group: *F*_3, 123_=2.70, *P*=.048; Control/High Engagers: Time: *F*_3, 117_=7.236, *P*<.001, Time × Group: *F*_3, 117_=2.698, *P*=.049). Between baseline and 18 weeks, Control group versus All Intervention group (Time × Group: *F*_1, 55_=5.721; *P*=.02) and Control group versus High Engagers group (Time × Group: *F*_1, 54_=6.029; *P*=.02) had significant changes over time in participants having discussions with their medical provider about their depression treatment goals with Control Group increasing over time and All Intervention and High Engagers decreasing over time (Table 3).

**Table 3.** Differences in treatment, treatment changes, and treatment-related goal setting between Control and All Intervention or High Engagers.

Question	Control, mean (SD)	All Intervention, mean (SD)	High Engagers, mean (SD)	Control/All Intervention Test	Control/High Engagers Test
Week 6					
Decision: Switched antidepressant medication^a^	0.03 (0.17)	0.21 (0.42)	0.25 (0.44)	*χ*^2^ (1, n=63)=5.4; *P*=.02	*χ*^2^ (1, n=59)=6.7; *P*=.01
Week 12					
Treatment option: Taking an antidepressant^a^	0.77 (0.43)	0.92 (0.27)	1.00 (0.00)	*χ*^2^ (1, n=61)=2.5; *P*=.11	*χ*^2^ (1, n=57)=5.9; *P*=.02
Decision: Started an antidepressant medication^a^	0.11 (0.32)	0.35 (0.49)	0.36 (0.49)	*χ*^2^ (1, n=61)=4.8; *P*=.03	*χ*^2^ (1, n=57)=5.0; *P*=.03
Decision: Decreased dose for an antidepressant medication^a^	0.00 (0.00)	0.12 (0.33)	0.14 (0.35)	*χ*^2^ (1, n=61)=4.2; *P*=.04	*χ*^2^ (1, n=57)=5.0; *P*=.03
Week 18					
Decision: Started an antidepressant medication^a^	0.06 (0.24)	0.25 (0.44)	0.27 (0.46)	*χ*^2^ (1, n=61)=4.3; *P*=.04	*χ*^2^ (1, n=55)=4.8; *P*=.03
Past 18 weeks, set any treatment goals for your depression^b^	1.34 (0.48)	1.14 (0.36)	1.09 (0.29)	*χ*^2^ (1, n=60)=3.2; *P*=.07	*χ*^2^ (1, n=54)=4.6; *P*=.03
Past 18 weeks, discussed depression treatment goals with your medical provider^b^	1.27 (0.45)	1.07 (0.26)	1.05 (0.21)	*χ*^2^ (1, n=61)=4.1; *P*=.04	*χ*^2^ (1, n=55)=4.6; *P*=.03

a 0 = No, 1 = Yes.

b 1 = Unsuccessful, 2 = Somewhat unsuccessful, 3 = Somewhat successful, and 4 = Very successful.

### Preliminary Results Significant Before but Not After Correcting for Multiple Comparisons

#### Shared Decision-Making

Reported use of SDM as measured by SDM-Q-9 was highest at baseline for all groups and decreased over the study period for all groups. Between baseline (B) and 6, 12, and 18 weeks, the SDM-Q-9 scores decreased significantly for both the Control group and the High Engagers group, but there were no differences in the amount of decrease between the 2 groups (B-6: *F*_1, 56_=4.527, *P*=.04; B-12: *F*_1, 54_=8.220, *P*=.006; B-18: *F*_1, 52_=6.559, *P*=.01). Scores also decreased significantly between baseline and 18 weeks for the Control group and All Intervention groups, with no difference between groups in the amount of decrease (*F*_1, 57_=6.075; *P*=.02; Table 2).

### Treatment Changes

#### Single Time Point

At 12 weeks, more participants in the High Engagers group were taking antidepressant medications than participants in the Control group (100% vs 77%; *χ*^2^_1_=5.9 [n=57]; *P*=.02; Table 3), but there was no difference between the All Intervention group and the Control group at this time point (92% vs 77%; *χ*^2^_1_=2.5 [n=61]; *P*=.11, Table 3). At 6 weeks, more participants in the All Intervention (7/34, 21%) and High Engagers (6/24, 25%) groups switched antidepressant medications than the Control group (1/35, 3%; Control/All Intervention: *χ*^2^_1_=5.4 [n=63]; *P*=.02; Control/High Engagers: *χ*^2^_1_=6.7 [n=59]; *P*=.01; Table 3).

At 12 weeks and at 18 weeks, both the All Intervention and High Engagers groups had a much larger percentage of participants starting an antidepressant medication than the Control group (Control/All Intervention at 12 weeks: 11% vs 35%, *χ*^2^_1_=4.2 [n=61]; *P*=.04; Control/All Intervention at 18 weeks: 6% vs 25%, *χ*^2^_1_=4.3 [n=61]; *P*=.04; Control/High Engagers at 12 weeks: 11% vs 36%, χ^2^_1_=5.1 [n=57]; *P*=.25; and Control/High Engagers at 18 weeks: 6% vs 27%, *χ*^2^_1_=4.8 [n=55]; *P*=.03; Table 3).

At 12 weeks, both the All Intervention and High Engagers groups had participants who decreased the dose in their antidepressant medication (12% and 14%, respectively), while the Control group had no participants decreasing their dose (All Intervention: *χ*^2^_1_=4.2 [n=61]; *P*=.04; High Engagers: *χ*^2^_1_=5.0 [n=57]; *P*=.03; Table 3).

#### Across Time Points

When compared over time, the All Intervention and High Engagers groups switched antidepressant medications at a higher rate than the Control group between baseline and 6 weeks (Control/All Intervention Time × Group: *F*_1, 60_=7.281, *P*=.009; Control/High Engagers Time × Group: *F*_1, 56_=7.995, *P*=.006) and across baseline, 6, and 12 weeks (Control/All Intervention Time × Group: *F*_2, 106_=3.232, *P*=.04; Control/High Engagers Time × Group: *F*_2, 104_=3.454, *P*=.04; Table 2). When observing changes over time between baseline and 12 or 18 weeks, all groups decreased the occurrence of starting an antidepressant medication at approximately the same rate (Control/All Intervention Time: *F*_3, 150_=3.905, *P*=.01; Control/High Engagers Time: *F*_3, 141_=3.817, *P*=.01). Over the course of the entire study, the Control group and High Engagers group showed a significant increase in discontinuing an antidepressant medication over time (*F*_3, 141_=3.285; *P*=.02). However, there were no significant differences between the groups themselves (*F*_3, 141_=1.992; *P*=.12).

### Goal Setting and Progress

At 18 weeks, more participants in the Control group (11/32, 34%) indicated that they set treatment goals for their depression in the last 18 weeks than those in the All Intervention (4/24, 14%; *χ*^2^_1_=3.2 [n=60]; *P*=.07) and High Engagers (2/22, 9%; *χ*^2^_1_=4.6 [n=54]; *P*=.03; Table 3) groups. More participants in the Control group (9/24, 27%) had also discussed their depression treatment goals with their medical provider within the last 18 weeks than those in the All Intervention (2/28, 7%; *χ*^2^_1_=4.2 [n=61]; *P*=.04) and High Engagers (1/22, 5%; *χ*^2^_1_=4.6 [n=55]; *P*=.03; Table 3) groups. At all time points compared with baseline, all participant groups set fewer goals for depression with no significant difference between groups (Table 3).

## Discussion

### Principal Findings

While we report on a small-scale pilot study that is underpowered for conclusive and generalizable conclusions, the data presented here can inform hypotheses for future, fully powered studies to examine the integration of digital resources into primary care practice. We evaluated a primary care-centric program designed to promote the use of digital tools and resources to promote patient or clinician communication and support SDM, goal setting, symptom monitoring, and other aspects of patient self-management. We found that some results held after controlling for multiple comparisons, while others did not and prompt the need for further exploration. Differences that held include that, compared with the control group, coordinated use of the program by both patients and clinical teams in primary care practices was associated with earlier communication between patients and medical providers and increased feelings of success in achieving goals. Significant differences at the individual analysis level that did not hold after correction included that intervention patient participants received earlier medication changes and reported starting a medication for depression, changing medications, or changing the dose of medication more frequently. For both intervention and control groups, reports of participating in SDM did not differ and were highest at baseline and decreased over time. Finally, improvement of depression symptoms was the same between the intervention and control groups. These findings indicate that there may be some benefits of the use of the app itself (eg, feelings of success in goal attainment) and the program overall (app plus clinical use of the program; eg, communication and treatment changes). However, this study was a pilot and was not adequately powered to be able to determine whether or not these differences would be generalizable.

### Use of Digital Tools to Support Communication and Shared Decision-Making

Despite recommendations and guidelines for its use, adoption of SDM is generally low in clinical care [19]. Here, we explored whether the coordinated use of the Primary Care Path program by patients and clinicians would facilitate and increase SDM in the intervention arm. We did not see a difference in reported quality of SDM between arms, but earlier communication between patients and clinicians and earlier treatment changes, potentially as a result of SDM, were noted in the intervention arm. While further exploration is needed, interviews with clinicians and patients in intervention practices indicated that they were using the program to inform treatment and management strategies [20]. SDM-Q-9 responses may not have shown differences for a few reasons: the program had no impact on SDM; the small scale of this study; the fact that both the control and intervention practices often participate in research projects, priming clinicians at all sites to be invested in quality improvement and implementation of SDM outside of their involvement in a given initiative or study [21]; and masking of program effects by confounding factors (eg, a multicenter study found that clinicians involved in committee work outside of medical practice do more SDM) [22]. More exploration of how or whether the program was used for SDM to support MDD management is warranted.

Interestingly, despite no difference in SDM being reported, we found that both All Intervention and High Engager groups reported decreasing conversations with their clinical teams over time, while Control patient participants reported increasing conversations over the study period. This held when correcting for multiple comparisons and suggests that All Intervention and High Engager groups may have addressed patient or provider communication needs earlier, while those without access to the Primary Care Path took longer to initiate or complete communication. These results suggest that the overall program may have facilitated or encouraged communication between the patient participants and their care teams, as we cannot tell whether the communication was initiated earlier by the intervention practice teams, the patients themselves, or both. It is noteworthy that conversations were occurring in both arms, but these conversations were not correlated to SDM, which decreased for all, as reported by SDM-Q-9 throughout the study.

### Goal Setting and Attainment and Feelings of Success

Despite the finding that goal setting increased more over time in the control group, analyses showed differences in rates of setting goals at 18 weeks, suggesting that using the app may have supported goal setting. This could be a feature of using any app aimed at aiding in self-management, although the Primary Care Path app specifically featured goal setting and provided a format to set and track goals. When patients have a way to record and track their goals, they may be more cognizant of their progress on those goals, thus explaining the finding of more self-reported success with goal attainment among the intervention group. Further exploration in a fully powered study is needed to better understand the role of the app and program on goal setting and attainment. However, our finding that both High Engagers and All Intervention participants reported feeling more successful in attaining their goals suggests that the coordinated use of the program by practices and their patients supported participants in recognizing their progress toward goals throughout their involvement in the study.

### Treatment Changes

Treatment changes (starting, switching, or decreasing the dose of an antidepressant) were more likely for participants in the intervention by All Intervention and High Engagers analyses at multiple time points, suggesting that the intervention may have facilitated medication management for depression treatment. Results indicating that communication may have been more frequent earlier for patients in the intervention arm may have played a role in earlier medication management. There is also evidence that symptom tracking by itself can positively influence health outcomes by increasing patient self-efficacy [23], and that patients who track their symptoms regularly and share their data with their clinician can feel that they receive more personalized, thus effective, care [24]. However, there is a lack of evidence on the effect of patient symptom tracking on treatment changes. It is also possible that recording symptoms and medication use made patients more aware of (1) day-to-day depression symptoms that might warrant a trial of medication and (2) side effects or other factors that might influence considering a change of medication. While communication differences were reported between arms, there was no difference in appointment frequency, so it seems likely that medication changes were made for specific reasons that were discussed during or between appointments.

It is unclear whether the positive outcomes related to symptom tracking that have been reported by similar studies are due to unreported treatment changes as a result of patient-recording data, or whether the data here are unique due to the features of the Primary Care Path program itself. The continuous nature of the symptom tracking in the app and the accessibility of this information to the clinician may have also facilitated earlier treatment changes by providing clinicians more evidence to necessitate treatment change. To our knowledge, at the time of this study, this was the only app focused on the management of MDD in primary care that automatically shares patient-reported data directly with their care team; however, further research is warranted to verify the effect and causality.

### Limitations

We acknowledge several study limitations. First, this is a pilot study and by nature includes nonrandomized practices and a small sample size, thus limiting the statistical power and increasing the potential for selection bias. Selection bias is possible with the small number of practices and sample size here. The goal of the study was to pilot-test the program for future use and garner data necessary to fully power a larger study where we can reduce selection bias from multiple fronts. The need for participants to own and be able to use a smartphone also affects the generalizability of our findings; however, smartphone ownership was at or near 85% during the course of this study, with rates above 60% for both low-income and older adults [25,26]. Another limitation is that the study design did not require clinician visits, so the use of or reference to patient-entered app content may have occurred in or outside the context of a clinic visit, thus impacting responses to the SDM-Q-9. Workflow changes were required for intervention practices, which may have introduced unknown factors that influenced the way the program was used. Examining variations in app use by patients may have been better addressed a priori to reduce the risk of type I error and reduce the potential for effects such as generalizability and error rate of the statistical tests. One of the most noteworthy limitations of the study is that it was initiated in the early phases of the COVID-19 pandemic, leading to circumstances that affected health care delivery and study implementation. Practice recruitment, staff engagement, and patient willingness to participate in a study may all have been affected by the implications of living through and providing medical care during the pandemic, although the pandemic may also have facilitated the adoption of the program and use of telehealth for this study. While these are problems that are commonly encountered in real-world studies, they also impact results, validity, and replicability. Limitations notwithstanding, this pilot study provided learning opportunities and findings that can be comprehensively examined in a fully powered study.

### Conclusions

This program, which facilitates clinician access to patient-generated goals and information, may enhance MDD management in primary care, leading to earlier medication optimization and patients feeling that they have achieved the treatment goals that matter most to them. This study contributes to beginning to understand the nature of coordinated use of digital tools by clinical practice teams and patients to improve outcomes for MDD in primary care. To clarify some conflicting findings and verify and determine the generalizability of results, a fully powered study with a larger sample of participants and practices is needed.

## Abbreviations

MDD: major depressive disorder
PHQ-9: Patient Health Questionnaire-9
SDM: shared decision-making
SDM-Q-9: Shared Decision-Making Questionnaire-9
